# Erlotinib is effective in pancreatic cancer with epidermal growth factor receptor mutations: a randomized, open-label, prospective trial

**DOI:** 10.18632/oncotarget.4216

**Published:** 2015-05-20

**Authors:** Jack P. Wang, Chen-Yi Wu, Yi-Cheng Yeh, Yi-Ming Shyr, Ying-Ying Wu, Chen-Yu Kuo, Yi-Ping Hung, Ming-Huang Chen, Wei-Ping Lee, Jiing-Chyuan Luo, Yee Chao, Chung-Pin Li

**Affiliations:** ^1^ Division of Gastroenterology, Department of Medicine, Taipei Veterans General Hospital, Taipei, Taiwan; ^2^ National Yang-Ming University School of Medicine, Taipei, Taiwan; ^3^ Division of Gastroenterology, Department of Internal Medicine, Renai Branch, Taipei City Hospital, Taipei, Taiwan; ^4^ Institute of Public Health, National Yang-Ming University, Taipei, Taiwan; ^5^ Department of Dermatology, Taipei Veterans General Hospital, Taipei, Taiwan; ^6^ Department of Dermatology, Heping Fuyou Branch, Taipei City Hospital, Taipei, Taiwan; ^7^ Department of Pathology, Taipei Veterans General Hospital, Taipei, Taiwan; ^8^ Division of General Surgery, Department of Surgery, Taipei Veterans General Hospital, Taipei, Taiwan; ^9^ Department of Medicine, National Yang-Ming University Hospital, Yilan, Taiwan; ^10^ Division of Oncology, Department of Medicine, Taipei Veterans General Hospital, Taipei, Taiwan; ^11^ Department of Medical Research, Taipei Veterans General Hospital, Taipei, Taiwan; ^12^ Department of Oncology Medicine, Taipei Veterans General Hospital, Taipei, Taiwan

**Keywords:** adenocarcinoma, pancreas, erlotinib, epidermal growth factor receptor

## Abstract

**Objective:**

To analyze the efficacy of gemcitabine with or without erlotinib for pancreatic cancer, and to determine the predictive role of epidermal growth factor receptor (*EGFR*) and *KRAS* mutations in these patients.

**Methods:**

This was a single-center, randomized, open-label, prospective trial. Eighty-eight chemotherapy-naïve metastatic pancreatic cancer patients were randomized for treatment with gemcitabine or gemcitabine plus erlotinib. *EGFR* and *KRAS* mutations were analyzed, respectively. The primary endpoint was the disease control rate.

**Results:**

Disease control rate (64% *vs.* 25%; *P* < 0.001), progression-free survival (median 3.8 *vs.* 2.4 months; *P* < 0.001), and overall survival (median 7.2 *vs.* 4.4 months; *P* < 0.001) were better in the gemcitabine plus erlotinib group than in the gemcitabine alone group. In the gemcitabine plus erlotinib group, disease control (85% *vs.* 33%; *P* = 0.001), progression-free survival (median 5.9 *vs*. 2.4 months; *P* = 0.004), and overall survival (median 8.7 *vs*. 6.0 months; *P* = 0.044) were better in patients with EGFR mutations than in those without EGFR mutations. KRAS mutation was not associated with treatment response or survival.

**Conclusions:**

Gemcitabine plus erlotinib is more effective than gemcitabine alone for treating metastatic pancreatic cancer patients, especially those with *EGFR* mutations. ClinicalTrials.gov number, NCT01608841.

## INTRODUCTION

Pancreatic cancer is a rapidly progressive malignancy with a 5-year survival rate of <10% [1]. Gemcitabine is the standard chemotherapy for metastatic pancreatic adenocarcinoma, but shows an objective response rate of only 5–10% and a median survival of 5.7–6.8 months [2-5]. During the last decade, many trials have been conducted on combined treatment with gemcitabine and targeted agents in an attempt to improve treatment outcome [6-8]. Unfortunately, most trials have failed to demonstrate any survival benefit compared to gemcitabine alone.

The mutation status of the *EGFR* tyrosine kinase (TK) domain is a predictive factor for EGFR inhibitor therapy in non-small cell lung cancer (NSCLC), especially among the Chinese people [9-14]. Whether this result applies to pancreatic cancer remains unclear. Erlotinib, a selective epidermal growth factor receptor (EGFR) tyrosine kinase inhibitor (TKI), is an orally active agent for advanced NSCLC and pancreatic cancer [3, 15]. The phase III NCIC CTG PA.3 trial comparing gemcitabine plus erlotinib and gemcitabine alone in advanced pancreatic cancer is the only trial of a targeted agent in pancreatic cancer that has shown a statistically significant improvement in survival [3]. However, the survival was only slightly longer for the gemcitabine plus erlotinib arm versus the gemcitabine plus placebo arm (median 6.24 *vs*. 5.91 months, *P* = 0.038). Considering cost effectiveness and the survival benefit, it is important to be able to accurately select the subgroup of patients who could benefit from this therapeutic regimen.

Pancreatic cancer shows the highest frequency of *KRAS* gene mutations among human cancers [16]. Ras signaling pathways are commonly activated in tumors and are involved in mediating the downstream effects of *EGFR* activation. Greater than 95% of these mutations involve codon 12 or 13 (exon 2), and a few involve codon 61 (exon 3) [16]. Whether *KRAS* mutations are associated with less efficient *EGFR*-directed targeted therapy in pancreatic cancer patients remains controversial and requires further investigation [17, 18].

To clarify the effects of adding erlotinib to gemcitabine and the roles of *EGFR* and *KRAS* mutations as predictive biomarkers in patients with advanced pancreatic cancer who receive these regimens, we performed this open-label, randomized, prospective study.

## RESULTS

### Patient characteristics

**Figure 1:**
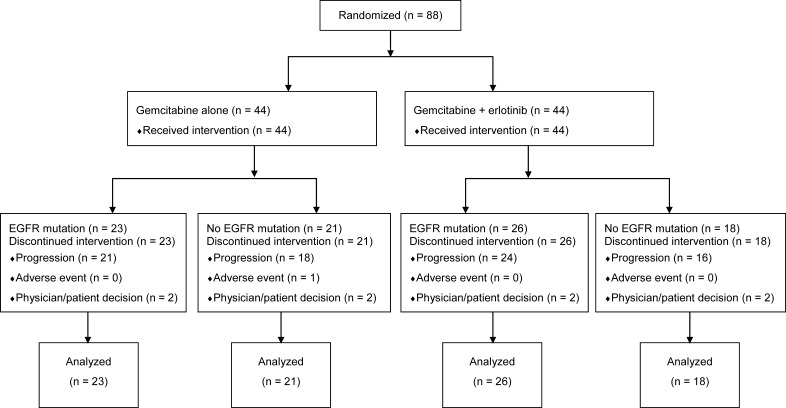
CONSORT diagram

Between July 2005 and June 2012, 88 patients were randomly assigned (44 gemcitabine plus erlotinib and 44 gemcitabine alone) (Figure 1). Baseline characteristics (Table 1) were well matched between the gemcitabine plus erlotinib arm and the gemcitabine alone arm. The median follow-up time was 7.2 months (range, 0.1–30.5 months) for the gemcitabine plus erlotinib group and 4.5 months (range, 0.4–16.9 months) for the gemcitabine alone group.

**Table 1:** Patient characteristics

Characteristics	All patients(n = 88)	Gemcitabine alone (n = 44)		Gemcitabine + erlotinib (n = 44)		P
		*EGFR* Mutation (+) (n = 23)	*EGFR* Mutation (−) (n = 21)	*EGFR* Mutation (+) (n = 26)	*EGFR* Mutation (−) (n = 18)	
Gender						0.711
Male	65 (74%)	16 (70%)	17 (81%)	20 (77%)	12 (67%)	
Female	23 (26%)	7 (30%)	4 (19%)	6 (23%)	6 (33%)	
Age (Years)						0.160
Median	70	67	74	69	65	
Range	33–91	37–86	33–91	35–86	43–84	
Performance status						0.057
0	73 (83%)	20 (87%)	16 (76%)	21 (81%)	16 (89%)	
1	13 (15%)	3 (13%)	5 (24%)	5 (19%)	0 (0%)	
2	2 (2%)	0 (0%)	0 (0%)	0 (0%)	2 (11%)	
Relapse after surgery	20 (23%)	4 (17%)	7 (33%)	5 (19%)	4 (22%)	0.593
Metastatic site						
Liver	61 (69%)	19 (83%)	14 (67%)	17 (65%)	11 (61%)	0.434
Peritoneum	49 (56%)	15 (65%)	10 (48%)	17 (65%)	7 (39%)	0.217
Lung	18 (20%)	2 (9%)	5 (24%)	6 (23%)	5 (28%)	0.423
Adrenal gland	1 (1%)	1 (4%)	0 (0%)	0 (0%)	0 (0%)	0.414
Bone	2 (2%)	0 (0%)	1 (5%)	1 (4%)	0 (0%)	0.609
Others	9 (10%)	3 (13%)	2 (10%)	2 (8%)	2 (11%)	0.939
Differentiation						0.479
Well	2 (2%)	0 (0%)	0 (0%)	1 (4%)	1 (6%)	
Moderately	18 (20%)	6 (26%)	6 (29%)	2 (8%)	4 (22%)	
Poorly	13 (15%)	4 (17%)	4 (19%)	2 (8%)	3 (17%)	
Unknown	55 (63%)	13 (57%)	11 (52%)	21 (81%)	10 (56%)	
CA19-9 (U/ml)						
Elevated (> 37)	71 (81%)	19 (83%)	19 (90%)	21 (81%)	12 (67%)	0.306
Median (range) in all patients	1,083 (2–1,439,400)	1,169 (4–10,411)	725 (14–97,622)	1,072 (8–439,380)	1,048 (2–1,439,400)	0.397

### Response and survival

**Figure 2:**
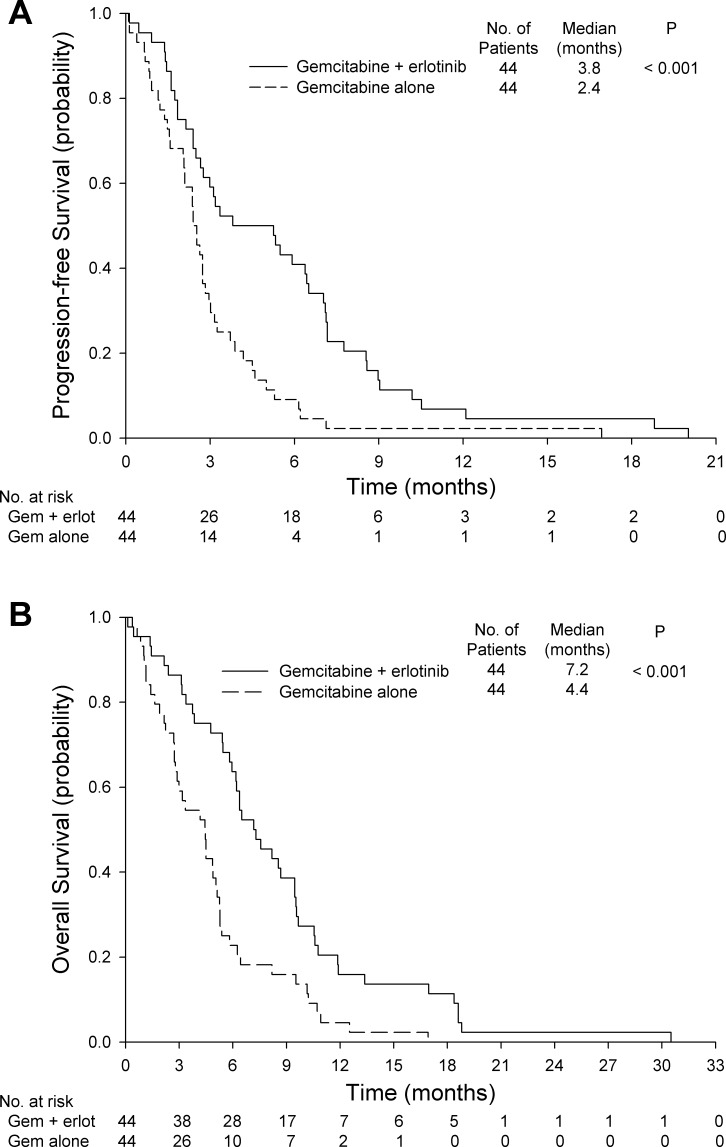
**A.** Progression-free survival for gemcitabine alone or gemcitabine plus erlotinib. **B.** Overall survival for gemcitabine alone or gemcitabine plus erlotinib. Gem: gemcitabine, Erlot: erlotinib.

The disease control rate (64% *vs*. 25%; *P* < 0.001), PFS (median 3.8 months; 95% confidence interval [CI], 1.2 to 6.4 months *vs*. 2.4 months; 95% CI, 1.9 to 2.9 months; *P* < 0.001), and OS (median 7.2 months; 95% CI, 5.3 to 9.0 months *vs*. 4.4 months; 95% CI, 3.0 to 5.9 months; *P* < 0.001) were significantly better in the gemcitabine plus erlotinib group than in the gemcitabine alone group (Table 2 and Figures 2A and 2B).

**Table 2:** Evaluation of the effect of *EGFR* mutations on treatment response

	Gemcitabine alone			Gemcitabine + erlotinib		
	Mutation (+) n = 23	Mutation (−) n = 21	P	Mutation (+)n = 26	Mutation (−)n = 18	P
Objective response						
Complete response	0/23 (0%)	0/21 (0%)		0/26 (0%)	0/18 (0%)	
Partial response	1/23 (4%)	3/21 (14%)		7/26 (27%)	1/18 (6%)	
Stable disease	4/23 (17%)	3/21 (14%)		15/26 (58%)	5/18 (28%)	
Progression of disease	16/23 (70%)	12/21 (57%)		2/26 (8%)	10/18 (56%)	
Not assessed*	2/23 (9%)	3/21 (14%)		2/26 (8%)	2/18 (11%)	
Response rate	1/23 (4%)	3/21 (14%)	0.335	7/26 (27%)	1/18 (6%)	0.115
Disease control rate	5/23 (22%)	6/21 (29%)	0.601	22/26 (85%)	6/18 (33%)	0.001
Best CA19-9 response						
> 80% decline	2/23 (9%)	4/21 (19%)	0.403	6/26 (23%)	2/18 (11%)	0.439
> 50% decline	6/23 (26%)	6/21 (29%)	0.853	15/26 (58%)	4/18 (22%)	0.020

* Included patients with either early death or symptomatic deterioration but no objective evaluation. Response rate included complete response and partial response. Disease control rate included complete response, partial response, and stable disease.

### *EGFR* mutation profile

Forty-nine (56%) patients had 62 mutations in *EGFR* exons 18–21 that caused substantial downstream amino acid changes (activating mutations, Supplementary Table 2). All mutations were heterozygous (Supplementary Figure 1) and confirmed by next-generation sequencing. Exon 20 was the most commonly mutated exon (50% of the mutations), followed by exon 19 (37%), exon 21 (10%), and exon 18 (3%). L778P in exon 20 was the most common mutation site (24% of all mutations), followed by K728R (19%) and W731X (13%) in exon 19, and I821T (15%) in exon 20. Patients with an L778P mutation who received gemcitabine plus erlotinib had a significantly higher disease control rate than those who received gemcitabine alone (71%, *N* = 7 *vs*. 0%, *N* = 8; *P* = 0.007, Supplementary Table 2).

Silent mutations were found in 18/88 patients. Six patients had a Q787Q mutation in exon 20, and two of these had additional missense mutations. Four had an F795F mutation, and two of these had other missense mutations. Two had a D800D mutation together with other missense mutations. One had a Q812Q mutation in exon 20 with a simultaneous missense mutation (I821T). Five had a D830D mutation in exon 21, and of these, one had a D830D mutation only. In both the gemcitabine alone and the gemcitabine plus erlotinib groups, response rates, disease control rates, PFS, and OS were not different between patients with silent *EGFR* mutations and patients without mutations. Patients with silent *EGFR* mutations were counted as patients without *EGFR* mutations in this study.

No activating *EGFR* mutation was found in the buffy coat of all patients, indicating that the mutations occurred somatically during carcinogenesis.

### *EGFR* mutations and tumor response

In the gemcitabine alone group, response rate, disease control rate, and CA19-9 response rate were comparable between patients with and without *EGFR* mutations (Table 2). In the gemcitabine plus erlotinib group, patients with *EGFR* mutations had a significantly higher disease control rate (85% vs. 33%; *P* = 0.001) and a significantly higher rate of a >50% CA19-9 decline (58% *vs*. 22%; *P* = 0.020).

### *EGFR* mutations and survival

**Figure 3:**
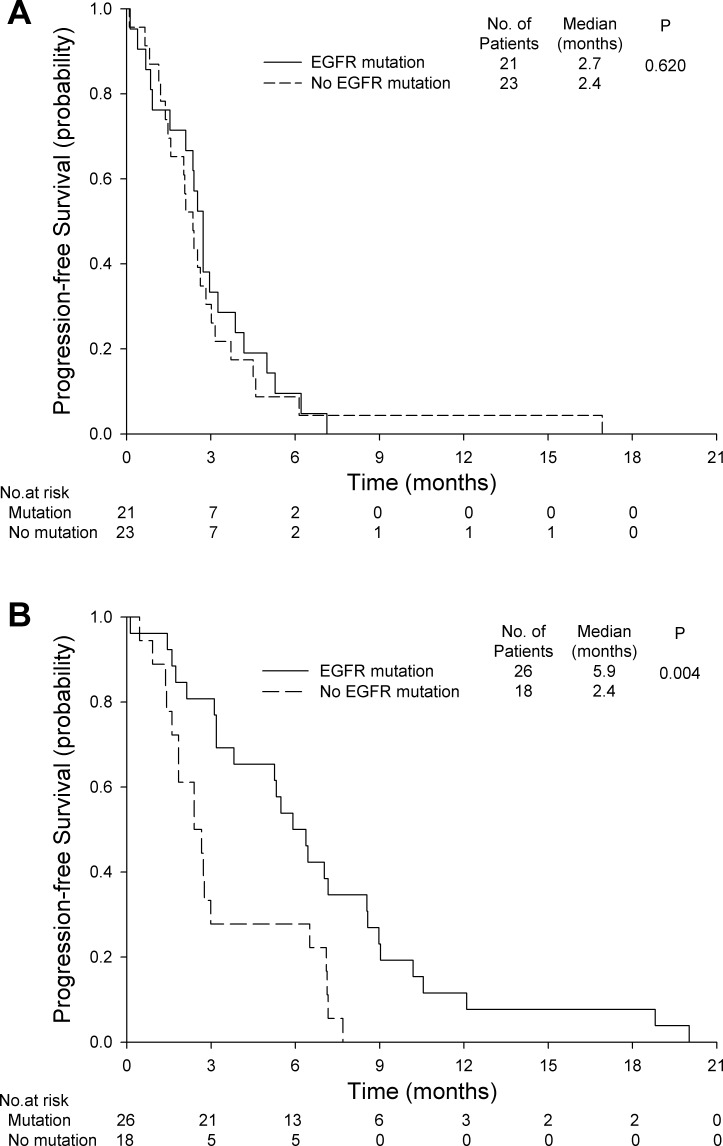
**Progression-free survival for A.** gemcitabine alone with or without *EGFR* mutations and **B.** gemcitabine plus erlotinib with or without *EGFR* mutations.

**Figure 4:**
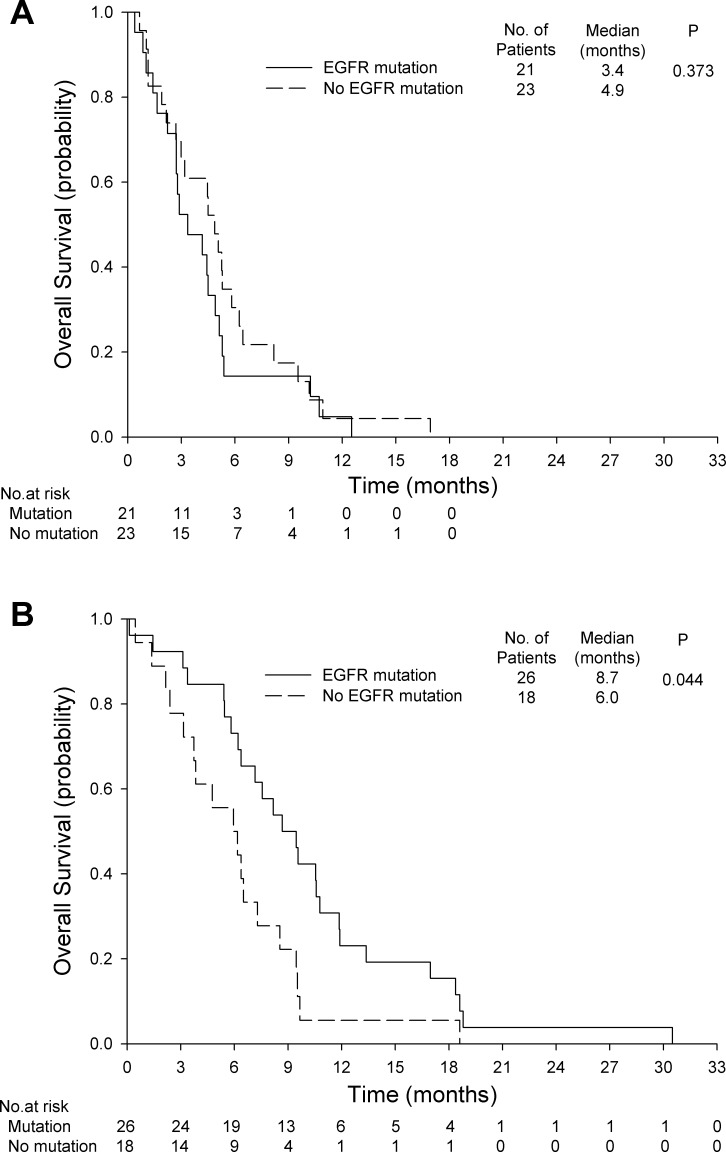
**Overall survival for A.** gemcitabine alone with or without *EGFR* mutations and **B.** gemcitabine plus erlotinib with or without *EGFR* mutations.

Patients receiving gemcitabine alone had similar PFS and OS regardless of the presence of *EGFR* mutations (Figures 3A and 4A). In the gemcitabine plus erlotinib group, patients with *EGFR* mutations had a significantly longer PFS (5.9 months; 95% CI, 4.5 to 7.3 months *vs*. 2.4 months; 95% CI, 1.5 to 3.3 months; *P* = 0.004, Figure 3B) and a significantly longer OS (8.7 months; 95% CI, 6.2 to 11.1 months *vs*. 6.0 months; 95% CI, 3.0 to 8.9 months; *P* = 0.044, Figure 4B) than those without *EGFR* mutations.

### *KRAS* mutations and treatment response

Tumor specimens from 83/88 pancreatic adenocarcinomas (94.3%) had a point mutation of *KRAS* in exon 2 or 3. There were 74 mutations in exon 2 (codon 12 or 13), two mutations in exon 3 (codon 61), and seven mutations in both exon 2 and exon 3. *KRAS* mutations were not associated with clinical and pathologic parameters including age, sex, ECOG performance status, tumor location, histologic differentiation, levels of CA19-9, response to chemotherapy, PFS, and OS. Patients with *KRAS* mutations did not have a statistically different rate of *EGFR* mutations (58% *vs*. 20%, *P* = 0.166) compared to patients without KRAS mutations.

### Toxicity and dosage modifications

Forty-four patients treated with gemcitabine and erlotinib and 44 treated with gemcitabine alone received at least one dose of study medication and were available for assessment of toxicity. Adverse events are summarized in Table 3.

**Table 3:** Adverse events

	Gemcitabine alone								Gemcitabine + erlotinib							
	Mutation (+) n=23				Mutation (−) n=21				Mutation (+) n=26				Mutation (−) n=18				
Grade	1	2	3	4	1	2	3	4	1	2	3	4	1	2	3	4
Haematological																
Leucopoenia	3	5	3	0	6	2	3	0	9	9	1	0	9	1	2	0
Neutropenia	2	1	3	0	2	2	2	2	7	2	4	3	3	0	2	2
Febrile neutropenia	0	1	0	0	2	1	0	0	4	1	0	0	2	0	0	0
Thrombocytopenia	13	2	2	0	12	3	0	0	13	3	1	0	8	2	4	0
Anaemia	7	12	3	0	3	12	3	0	5	14	5	0	3	12	2	0
Non-haematological																
Nausea	3	2	0	0	3	2	0	0	3	1	1	0	3	2	1	0
Vomiting	3	3	1	0	2	3	0	0	5	0	1	0	2	4	0	0
Diarrhoea	1	0	0	0	4	0	0	0	2	2	0	0	2	0	1	0
Elevated level of alanine aminotransferase	8	2	2	0	7	0	0	1	13	4	2	0	6	1	2	0
ILD-like syndrome	0	0	0	0	0	0	0	0	0	0	1	0	0	0	0	0
Fever	1	1	0	0	3	1	0	0	6	3	0	0	4	1	0	0
Rash	0	1	0	0	0	1	0	0	12	6	1	0	5	1	2	1
Stomatitis/mucositis	0	1	0	0	1	0	0	0	2	1	0	0	3	1	0	0

Treatment was generally well tolerated in both arms. Patients receiving gemcitabine plus erlotinib experienced a significantly higher frequency of rash (64% *vs*. 2%, *P* < 0.001), but these were generally grade 1 or 2. The incidence of other adverse events, including hematologic toxicity and elevations in alanine aminotransferase, were similar between groups. There were no protocol-related deaths. One patient in the gemcitabine plus erlotinib group had interstitial lung disease (ILD)-like syndrome possibly related to therapy.

The gemcitabine dose intensity was similar in the gemcitabine alone and gemcitabine plus erlotinib groups (905 ± 182 mg•m^−2^•dose^−1^ × 8.2 ± 5.5 doses *vs*. 881 ± 236 mg•m^−2^dose^−1^ × 17.1 ± 13.4 doses). Four (9%) patients receiving erlotinib and gemcitabine had at least one dose reduction of their oral agent. The erlotinib dose intensity was 96.8 ± 12.6 mg•m^−2^day^−1^ × 17.9 ± 13.7 weeks.

### Skin rash and survival in the gemcitabine plus erlotinib group

**Figure 5:**
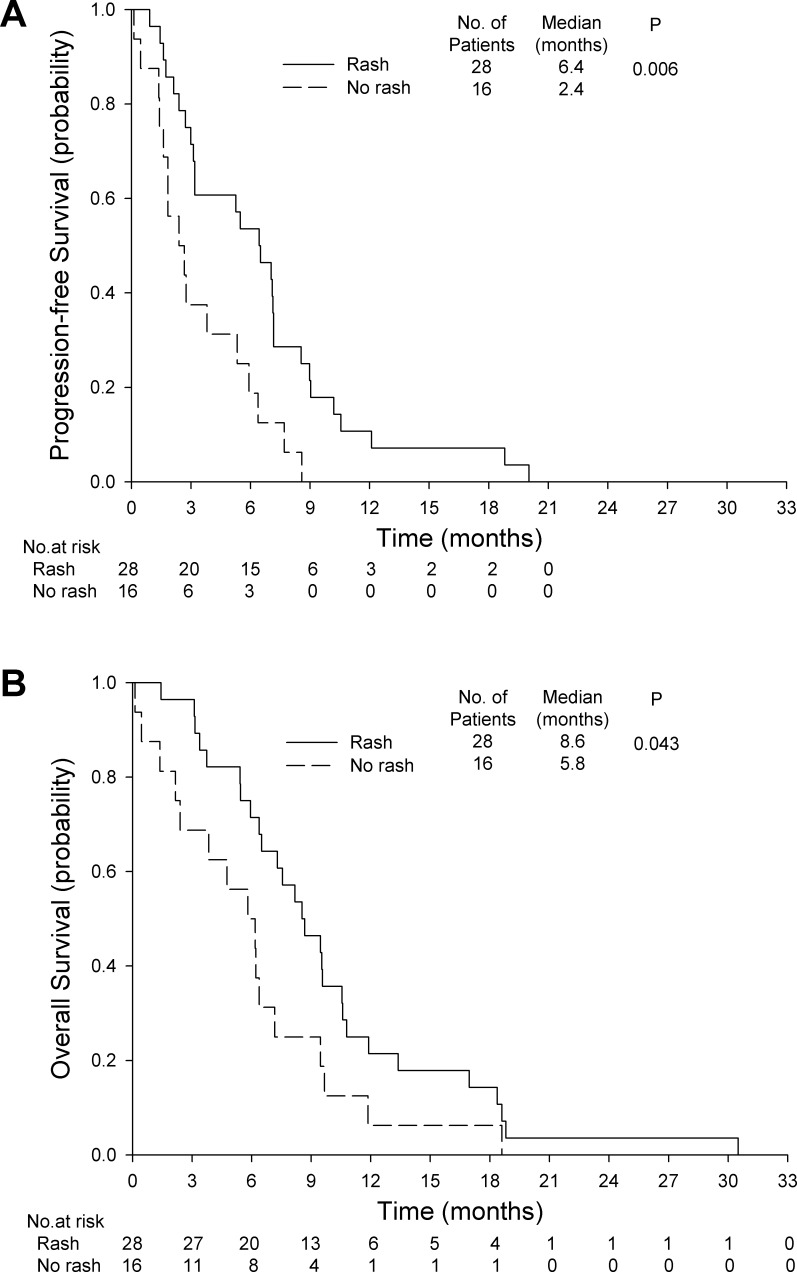
Skin rash and survival in the gemcitabine plus erlotinib group **A.** progression-free survival **B.** overall survival.

Of the 44 patients receiving gemcitabine plus erlotinib, 16 had no rash, 17 had grade 1 rash, 7 had grade 2 rash, 3 had grade 3 rash, and 1 had grade 4 rash. Patients with *EGFR* mutations did not have a significantly different rate of skin rash than patients without *EGFR* mutations (73% *vs*. 50%; *P* = 0.118). The presence of rash was associated with significantly higher rates of disease control (75% *vs*. 44%; *P* = 0.038) and treatment response (29% *vs*. 0%; *P* = 0.036). Patients with skin rash had significantly longer PFS (6.4 months; 95% CI, 4.1 to 8.7 months *vs*. 2.4 months; 95% CI, 0.8 to 4.0 months; *P* = 0.006, Figure 5A) and OS (8.6 months; 95% CI, 6.1 to 11.0 months vs. 5.8 months; 95% CI, 3.1 to 8.6 months; *P* = 0.043, Figure 5B).

## DISCUSSION

*EGFR* mutations are known to be the most important determinants of NSCLC patient response to EGFR-TKIs [9-14]. However, the importance of *EGFR* mutations in pancreatic cancer has not been investigated until now. The *EGFR* mutation profile in the Chinese population analyzed in this study can be used to predict treatment response and survival in pancreatic cancer patients receiving gemcitabine plus erlotinib.

The NCIC CTG PA.3 trial demonstrated the beneficial effects of erlotinib in the treatment of pancreatic cancer [3]. However, no Chinese patients were included in that study and the prolongation of median OS with erlotinib was only slightly longer, at 0.33 months. Neither EGFR protein expression status nor gene copy number was identified as a marker predictive of survival benefit following combination treatment with erlotinib and gemcitabine [3, 17]. In the present study, we used direct sequencing to detect *EGFR* mutations and confirmed by next-generation sequencing, which accurately predicted treatment response and survival in Chinese pancreatic cancer patients.

The median OS of patients treated with gemcitabine plus erlotinib (regardless of *EGFR* mutations) was 7.2 months. The NCIC CTG PA.3 trial, which included both locally advanced pancreatic cancer and metastatic pancreatic cancer patients, showed that the median OS with gemcitabine plus erlotinib was 6.24 months [3]. The more favorable outcome is likely because of the higher rate of *EGFR* mutations in this study [19, 20], which enhanced treatment efficacy [9, 10, 12, 13]. These results are encouraging, especially because all patients were already in the metastatic stage.

The median survival with gemcitabine alone in this trial (4.4 months) is lower than that found by Burris et al. (5.65 months) [2], Moore et al. (5.91 months) [3], Conroy et al. (FOLFIRINOX, 6.8 months) [4], and the Metastatic Pancreatic Adenocarcinoma Clinical Trial (MPACT, 6.6 months) [5]. In this trial, the disease control rate with gemcitabine alone (25%) is also lower than that in the gemcitabine arms of the above trials (Burris et al., 44.4%; Moore et al., 49.2%; FOLFIRINOX, 50.9%; MPACT, 33%) [2-5]. Our results are similar to another study of patients of Chinese descent (median survival 4 months) [21]. Other than ethnic differences, advanced age (median age 70 years in this study vs. 61-64 years in other studies) and advanced stage (all stage IV) may also account for the relatively poor survival and lower disease control rate in the gemcitabine alone group in this study.

In NSCLC and breast cancer, the prevalence of *EGFR* mutations between Caucasian and Asian patients is different [10, 22]. For NSCLC, there are variations between Chinese and other Asian populations [23, 24]. Lynch et al. did not detect any mutations in *EGFR* exons 19 and 21 in 40 pancreatic cancer primary tumors in Caucasian patients [9]. Tzeng et al. reported that 6/9 pancreatic cancer cell lines and 25/31 Caucasian pancreatic cancer patients had *EGFR* mutations, but all were silent mutations [20]. Oh et al. and Lee et al. showed a rate of 1.5–2.5% *EGFR* mutations in Korean pancreatic cancer cohorts [16, 25]. However, none of these patients was Chinese. Our study was conducted in Taiwan, where the majority of the population is of Chinese descent, and the pancreatic cancer *EGFR* mutation rate was 56%. Improvement in OS for pancreatic cancer patients treated with erlotinib was apparent in our study because of the high proportion of cases with *EGFR* mutations compared to other studies. Currently, pancreatic cancer is the 7th leading cause of cancer death among the Chinese, making *EGFR* mutation detection an important issue in the treatment of pancreatic cancer [26].

The mutation sites in Chinese pancreatic cancer patients are also different from those reported in other populations [16, 25]. The presence of *EGFR*-activating mutations in Chinese patients emphasizes ethnic, geographic, and environmental variations in pancreatic cancer mutation profiles that could result in different responses to erlotinib treatment. Similar to reports by Tzeng et al. and Lee et al., we found Q787Q to be the most common silent mutation [16, 20]. In addition, we found four silent mutations that have not yet been reported, suggesting that *EGFR* silent mutations may vary among ethnicities.

The pattern of *EGFR* mutations varies among different solid tumors. In NSCLC, missense L858R and in-frame deletion E746_A750del constitute 78.2% of mutations [27]. All mutations in exon 19 (7.3%) were in-frame deletions (E746_A750del) in the squamous cell carcinoma of the head and neck [28], whereas two types of deletions, K745_E749del and E746_A750del, were found in exon 19 in cholangiocarcinomas [29]. Substitutions in exons 19 (E749K) and 20 (E762G and A767T) were predominant in 12% of colorectal carcinomas [30]. All of these mutations are different from those found in our work. Owing to these differences in the mutation profiles of *EGFR* for pancreatic cancer, compared with other cancers, commercially available *EGFR* mutation detection kits cannot replace direct sequencing [31].

Erlotinib-mediated disease control in *EGFR*-mutated pancreatic cancer patients is modest, with many patients achieving stable disease (85%). This is similar to the observations of Kwak et al. [19] who showed that both pancreatic cancer patients with *EGFR* mutations exhibited disease stabilization in response to therapy with erlotinib and capecitabine; however, this result does not fall within the same category of dramatic responses seen in NSCLC [9-14]. Further investigation is required to determine whether the site of primary cancer accounts for the disparate effects of TKI.

The gemcitabine plus erlotinib group had a higher disease control rate than the gemcitabine alone group for patients with an L778P mutation. Other mutation sites failed to demonstrate a significant difference in response and disease control rates. Because of their rarity, it is difficult to draw definitive conclusions regarding the true relationship between uncommon *EGFR* mutations and sensitivity to erlotinib. Further studies with larger patient numbers and functional analysis of the *EGFR* mutations are warranted to elucidate the impact of the individual mutation sites on the efficacy of erlotinib use.

There was no correlation between *KRAS* and *EGFR* mutations in our study, unlike the case in lung cancer, where these are generally mutually exclusive [32]. Furthermore, *KRAS* mutation status did not predict how patients would respond to erlotinib in our study, which is in line with the pivotal PA.3 study [17]. The small numbers of patients in our study (5.7%) who had wild-type KRAS make comparisons difficult with data from other randomized trials.

Most adverse events associated with erlotinib plus gemcitabine treatment in this study were mild-to-moderate, consistent with previous studies [2-5]. Rash was more frequent with erlotinib plus gemcitabine than with gemcitabine alone, and we observed the association between rash and a better outcome that has been seen in other pancreatic cancer studies of EGFR inhibitors [3, 33, 34]. One patient in the gemcitabine plus erlotinib arm had ILD-like syndrome. Gemcitabine and EGFR TKIs are both known to cause ILD-like syndrome, and it is possible that there is a more-than-additive effect when these agents are combined [35]. The incidence seen in this study (2.3%) is similar to that in other trials with combined gemcitabine and erlotinib [3].

FOLFIRINOX (a multidrug regimen of fluorouracil, leucovorin, irinotecan, and oxaliplatin) showed an increased median survival of 4.3 months but a worse safety profile compared to treatment with gemcitabine [4]. In another phase III multinational MPACT study, the combination of gemcitabine and nab-paclitaxel was shown to be superior to gemcitabine monotherapy, with a 1.8-month increase in median survival [5]. Combination therapy with gemcitabine plus erlotinib, gemcitabine plus nab-paclitaxel, or leucovorin, fluorouracil, irinotecan, and oxaliplatin is now considered first-line chemotherapy for metastatic pancreatic cancer.

Pancreatic cancer tends to be relatively resistant to chemotherapy and targeted therapy [6-8], and only a few regimens have proven efficacious [2-5, 36]. Our study first found that *EGFR* mutations are common in Chinese pancreatic cancer patients. In addition, this study showed that *EGFR* mutations can predict gemcitabine plus erlotinib treatment response and survival. However, this study did have some limitations. First, the sample size of 88 participants is relatively small. However, a strong association was still detected between *EGFR* mutations and the efficacy of erlotinib in pancreatic cancer. Second, all enrollees were Chinese, and these results might not apply to other ethnicities. These issues require further investigation in additional larger clinical studies.

In conclusion, this is the first study to show a significant association between *EGFR* mutations and survival benefit in metastatic pancreatic cancer patients receiving erlotinib combined with gemcitabine. Detection of *EGFR* mutations can be used to identify the subgroup of patients with pancreatic cancer in whom *EGFR* may be essential for tumor growth and who would thus benefit from treatment with erlotinib.

## MATERIALS AND METHODS

### Patient selection

This study was a single-center, randomized, open-label, prospective phase II trial. Inclusion criteria were metastatic pancreatic adenocarcinoma, presence of at least one measureable lesion, Eastern Cooperative Oncology Group (ECOG) performance status ≤ 2, absolute neutrophil count ≥ 1,500/mm^3^, platelet count ≥ 100,000/mm^3^, serum creatinine level ≤ 1.5× upper normal limit, aspartate aminotransferase or alanine aminotransferase level ≤ 5× upper normal limit, adequate samples for histological and mutational analyses, and absence of any other malignancy or serious medical or psychological illness that would preclude informed consent.

### Ethics statement

Investigation has been conducted in accordance with the ethical standards and according to the Declaration of Helsinki and according to national and international guidelines and has been approved by the Institutional Review Board of Taipei Veterans General Hospital, conforms to CONSORT guidelines, and was registered as ClinicalTrial.gov NCT01608841.

### Randomization

Patients were randomly allocated in a 1:1 ratio to receive either gemcitabine (Gemzar ^®^; Eli Lilly and Company, Indianapolis, IN, USA) plus erlotinib (Tarceva; OSI Pharmaceuticals, Farmingdale, NY, USA) or gemcitabine alone using a computerized random number function in Microsoft Excel (Microsoft, Redmond, WA, USA). A block randomization procedure was employed to ensure equal group allocation.

### Treatment

Gemcitabine 1,000 mg/m^2^ was infused over 30 minutes on days 1, 8, 15, 22, 29, 36, and 43 followed by a 1-week rest in cycle 1, and on days 1, 8, and 15 in all subsequent 4-week cycles. Erlotinib was taken orally at 100 mg once a day. Treatment continued until disease progression or intolerable toxicity.

### Pathology review, buffy coat, and DNA preparation

Pathological specimens were obtained on initial diagnosis from sonography-guided (40 patients) or computed tomography-guided biopsy (15 patients), endoscopic biopsy (4 patients), open biopsy (9 patients), or pancreatectomy (20 patients). Tumor samples were obtained from primary pancreatic tumor (*n* = 44) or metastatic tissue (*n* = 44). All tissue sections were cut from formalin-fixed paraffin-embedded tumor blocks, and histological examination was performed by a pathologist (YCY) blinded to clinical information. All tumor regions were confirmed prior to DNA isolation. The buffy coat, representing the normal tissue and separated using Histopaque solution (Histopaque-1119; Sigma-Aldrich, St. Louis, MO, USA), was obtained from all patients at enrollment. DNA was extracted from sections and the buffy coat using the QIAamp DNA Mini kit (Qiagen, Hilden, Germany).

### Polymerase chain reaction (PCR) and sequencing of the EGFR gene

Eight pairs of oligonucleotide primers were used to amplify exons 18–21 of the *EGFR* gene using PCR as described previously (Supplementary Table 1) [9]. The PCR products were extracted and purified from a 1.5% agarose gel using the QIAquick gel extraction kit (Qiagen) and sequenced using the ABI PRISM BigDye Terminator Cycle Sequencing Ready Reaction kit and the ABI PRISM 3700 DNA Analyzer (PE Applied Biosystems, Foster City, CA, USA). PCR amplicon sequences were compared with the *EGFR* cDNA sequence obtained from Genbank (accession no. GI:22022643) by using Mutation Surveyor™ 3.0 (SoftGenetics, State College, PA, USA) and manual review. All sequence variants were verified by reverse sequencing.

### Ion torrent deep-amplicon sequencing

To eliminate PCR artifacts and false positives, the GeneRead DNA FFPE kit was used to extract DNA. The DNA extracted from FFPE was used for multiplex PCR of a panel covering 18–21 exons in the *EGFR* gene. Fragment libraries were constructed using DNA fragmentation, barcode and adaptor ligation, and library amplification, according to the manufacturer's instructions, as stipulated in the Ion Xpress Plus Fragment Library Kit (Life Technologies, Grand Island, NY, USA). Size distribution of the DNA fragments was analyzed using the Agilent Bioanalyzer with the High Sensitivity Kit (Agilent, Santa Clara, CA, USA). Template preparation, emulsion PCR, and Ion Sphere Particle (ISP) enrichment was performed using the Ion PGM Template OT2 200 Kit (Life Technologies, Grand Island, NY, USA) according to the manufacturer's instructions. The ISPs were loaded onto a 314 chip and sequenced using an Ion PGM Sequencing 200 Kit v2 (Life Technologies, Grand Island, NY, USA).

### Bioinformatics analysis

After a successful sequencing reaction, the raw signal data were analyzed using Torrent Suite v4.0.2. The pipeline includes signaling processing, base calling, quality score assignment, adapter trimming, PCR duplicate removal, read alignment to human genome19 reference, mapping quality QC, coverage analysis, and variant calling. After completion of the primary data analysis, lists of detected sequence variants (SNVs and INDELs) were compiled in the VCF (Variant call file) format. For downstream analysis, variants with a minimum coverage of 500 reads were selected.

### PCR and sequencing of the KRAS gene

The cobas^®^ KRAS Mutation Test (Roche Diagnostics, Basel, Switzerland) was used according to the manufacturer's protocol [37]. Samples (50 ng of DNA) were aliquoted in 96-well plates, and negative and positive controls from the kit were added. Data were automatically processed by cobas^®^ software.

### Evaluation of treatment response and safety

Objective tumor response was evaluated every 8 weeks or earlier for patients with suspected progression according to the Response Evaluation Criteria in Solid Tumors (RECIST 1.0) [38]; responses were confirmed at least 28 days after the first documentation of a complete or partial response or stable disease. Images were blindly reviewed by a protocol-trained radiologist (RCL). Serial measurements of carbohydrate antigen 19-9 (CA19-9) were performed at baseline and every 4 weeks thereafter. Safety was monitored by assessing treatment-related adverse events and serious adverse events according to the National Cancer Institute Common Terminology Criteria for Adverse Events (v3.0).

### Statistical analysis

A superiority test was conducted with the assumption that gemcitabine plus erlotinib would have a higher disease control rate [3], defined as the best overall response for complete or partial response or stable disease, than gemcitabine alone. The disease control rate was estimated as 30% in the gemcitabine alone group, and 60% in the gemcitabine plus erlotinib group [5, 39]. A sample size of 44 for each group was required for 80% power with 5% error (two-tailed) and 5% loss to follow-up [40]. Secondary outcome measures were response rate, progression-free survival (PFS), overall survival (OS), relation to *EGFR* and *KRAS* mutation status, and safety. PFS was defined as the time from random assignment to disease progression or death as a result of any cause. For patients alive without documented disease progression at the time of the analysis, PFS was censored at the time of the last tumor assessment. If no post-baseline assessment was performed, the date of random assignment plus 1 day was used as the censor date. OS was defined as the time from random assignment until death as a result of any cause, and patients alive at the time of the analysis were censored at the date of last contact. In the response rate analysis, patients without a post-baseline assessment were not assessed. PFS and OS were calculated using the Kaplan-Meier method and log-rank tests. The Student's t test or one-way analysis of variance was used to analyze continuous variables between groups. The association between skin rash, *EGFR* mutations, response rate, and disease control rate were compared using the Chi-square test or Fisher's exact test. Statistical analyses were performed using SPSS software (SPSS 21.0, SPSS Inc., Chicago, IL, USA). *P* < 0.05 was considered statistically significant.

## SUPPLEMENTARY MATERIALS FIGURE AND TABLES


