# Supplementary material for: Erlotinib is effective in pancreatic cancer with epidermal growth factor receptor mutations: a randomized, open-label, prospective trial
Source: Oncotarget. 2015 May 20;6(20):18162–73. doi: 10.18632/oncotarget.4216 (PMC4627242; doi:10.18632/oncotarget.4216)
Supplement: Supplementary file 1 [file oncotarget-06-18162-s001.pdf]

Erlotinib is effective in pancreatic cancer with epidermal growth factor receptor mutations: a randomized, open-label, prospective trial

Supplementary Material

Supplementary Figure 1. DNA sequencing analysis of *EGFR* gene from exons 18 to 21.

Exon 18

(A) Normal sense (B) Tumor sense (C) Tumor antisense

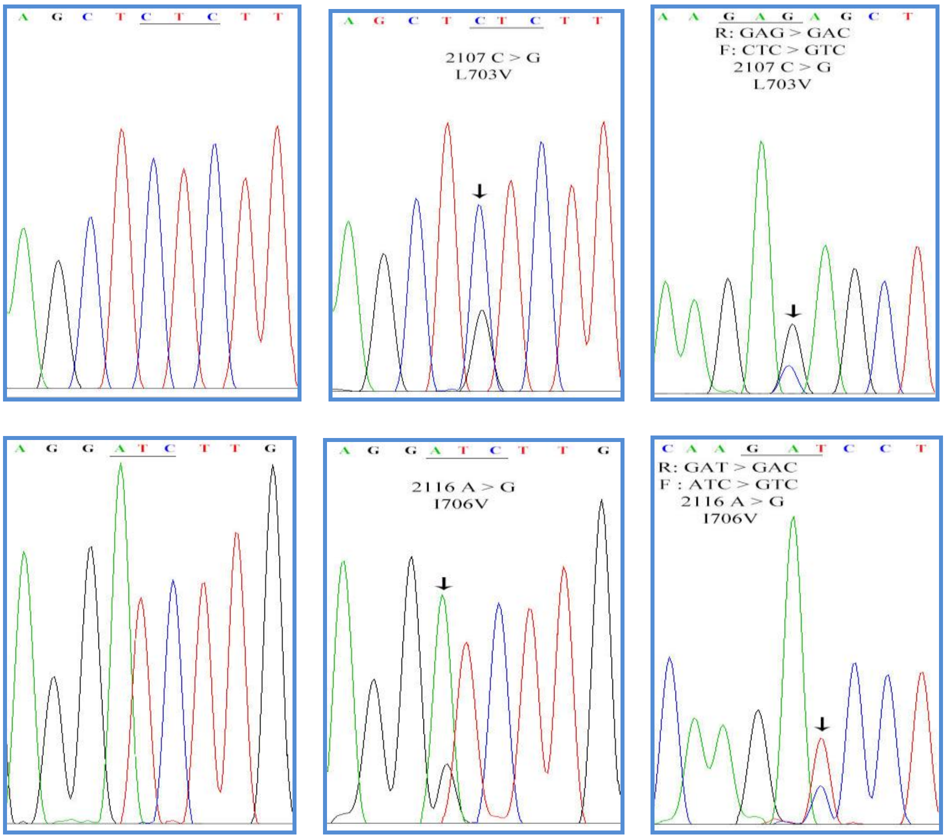

Exon 19

(A) Normal sense (B) Tumor sense (C) Tumor antisense

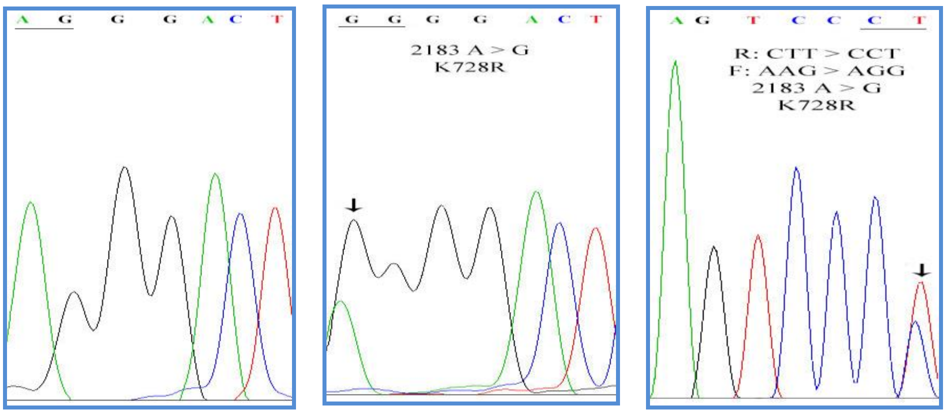

*The peak of 2182A was not shown due to its position at exon 18.

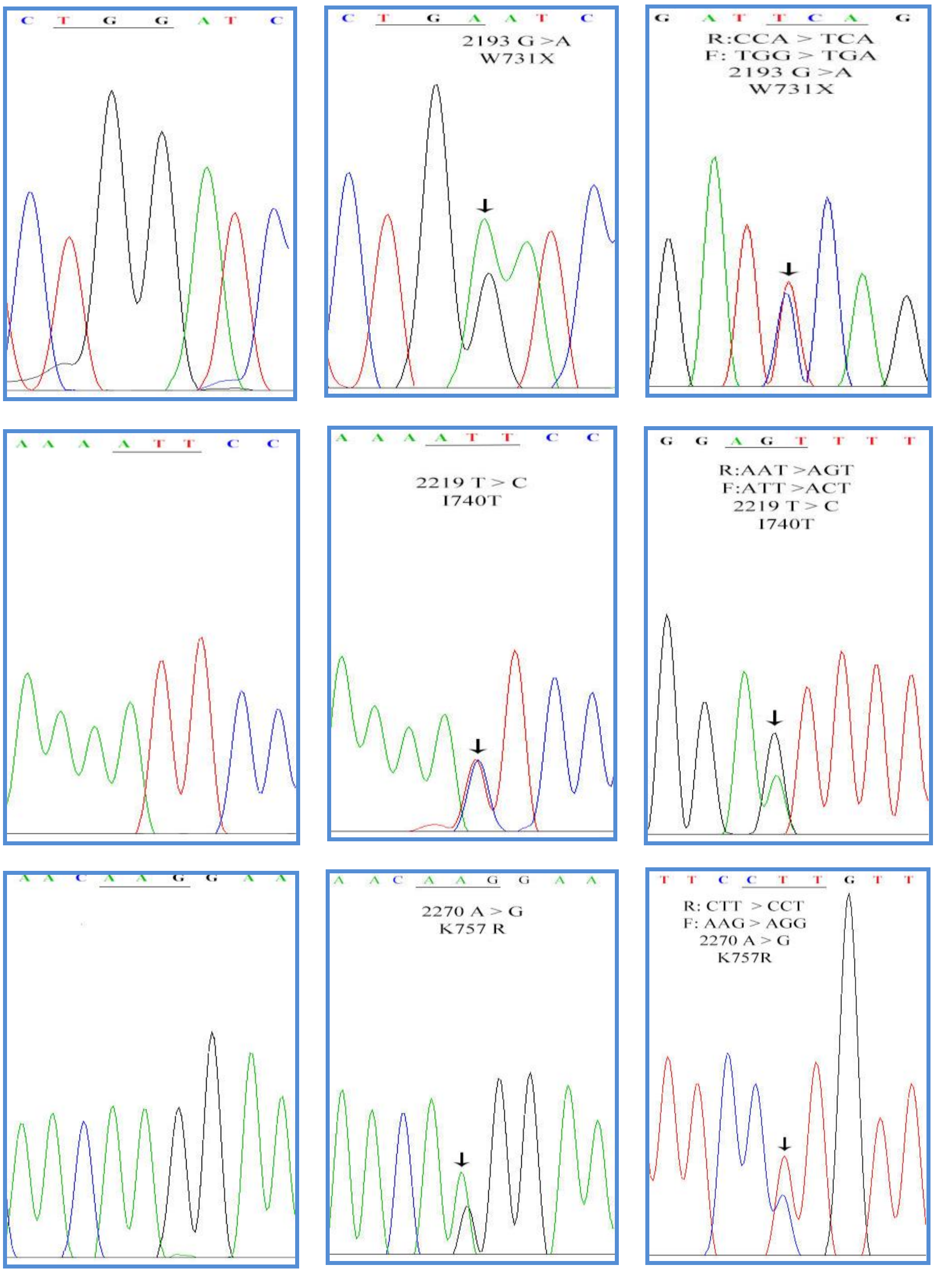

Exon 20

(A) Normal sense (B) Tumor sense (C) Tumor antisense

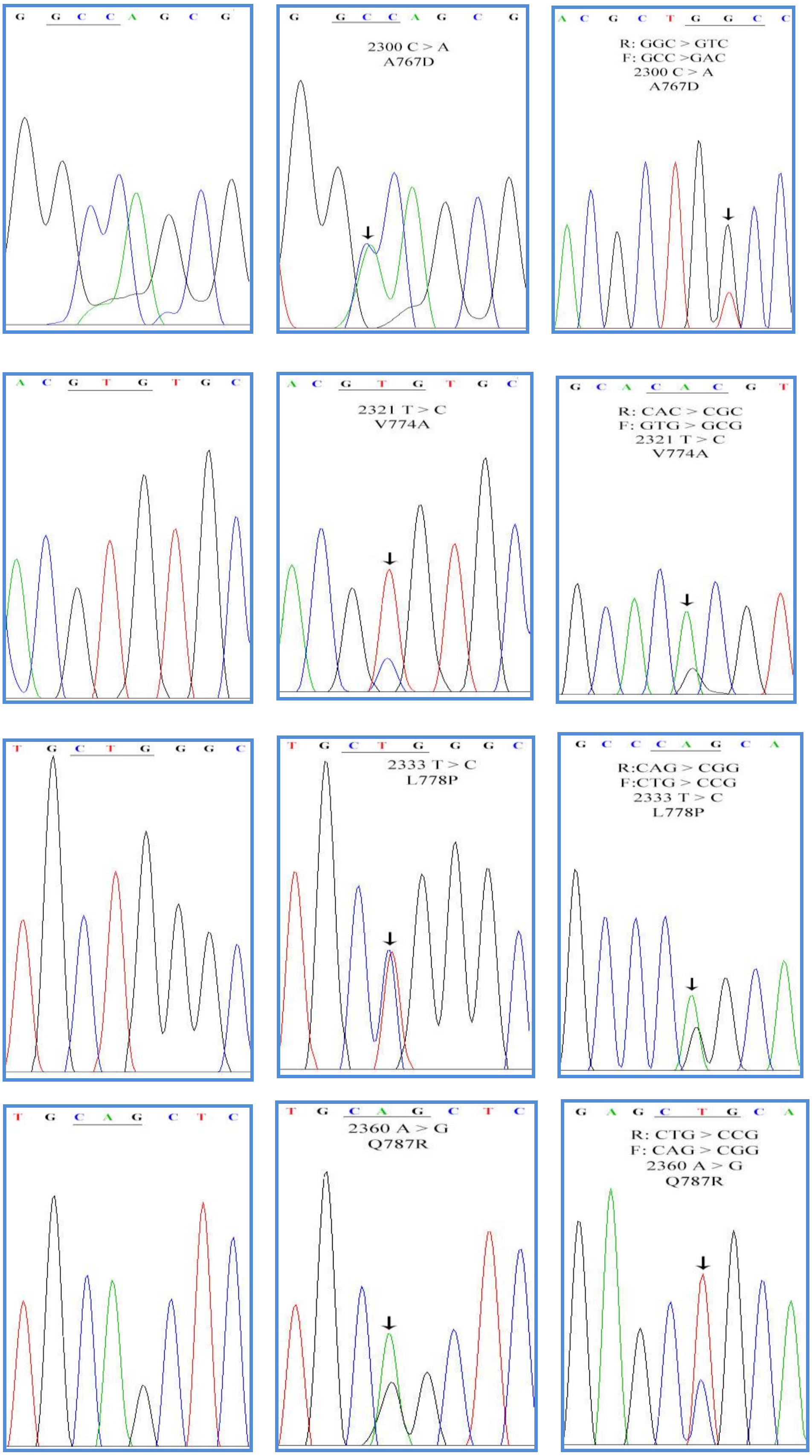

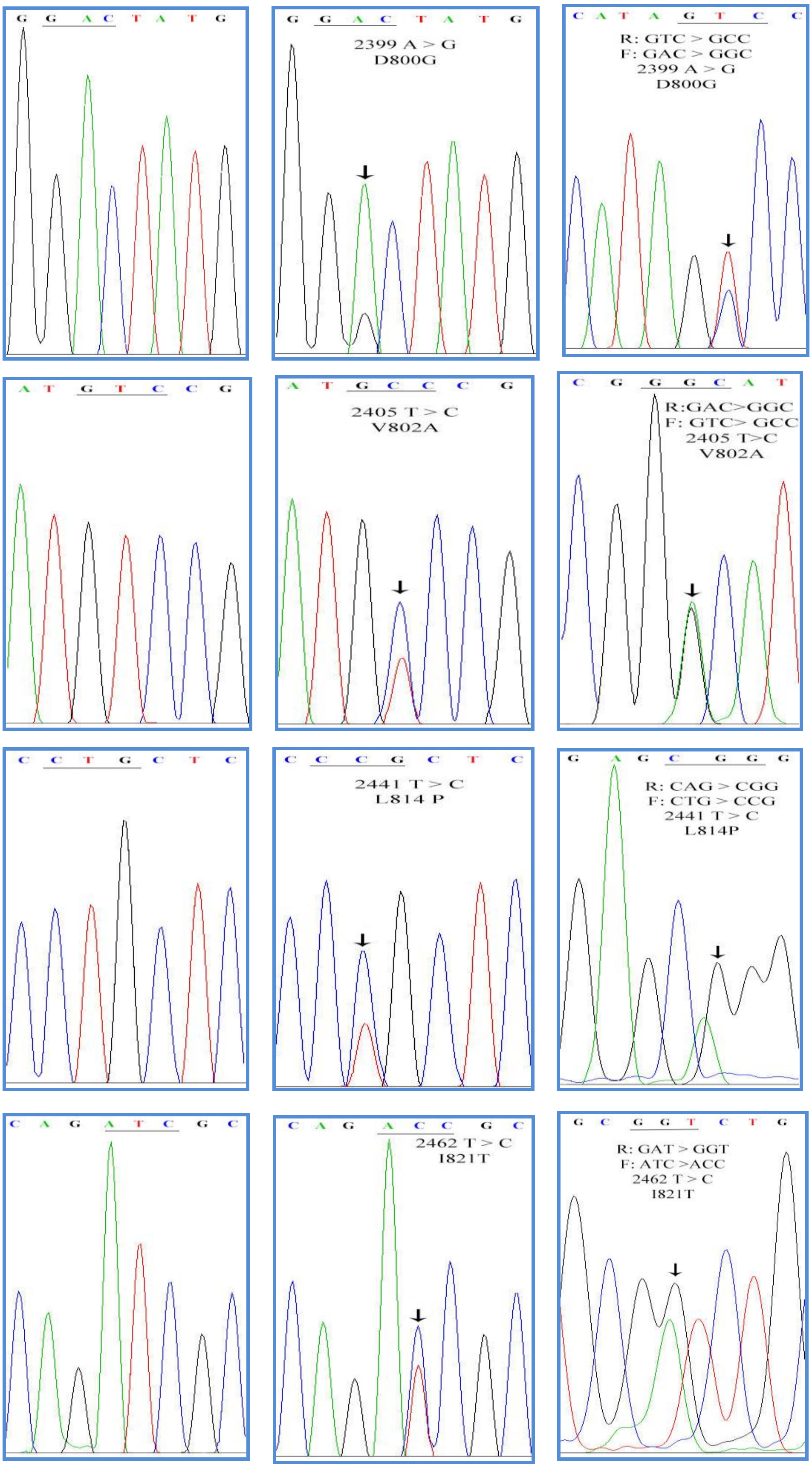
2399 A > G
D800G
R: GTC > GCC
F: GAC > GGC
2399 A > G
D800G
2405 T > C
V802A
R:GAC>GGC
F: GTC> GCC
2405 T>C
V802A
2441 T > C
L814 P
R: CAG > CGG
F: CTG > CCG
2441 T > C
L814P
2462 T > C
I821T
R: GAT > GGT
F: ATC >ACC
2462 T > C
I821T

Exon 21

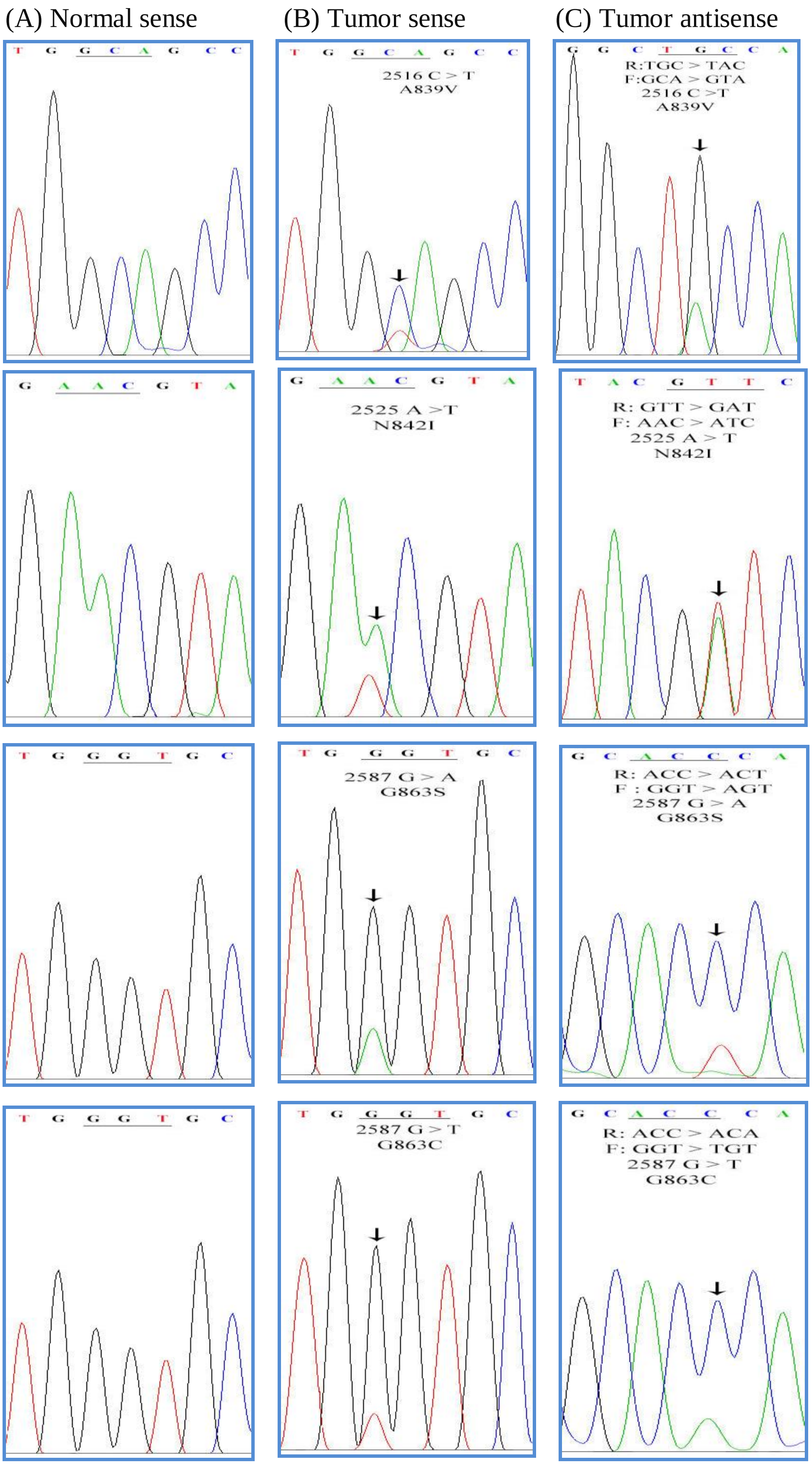
(A) Normal sense
(B) Tumor sense
(C) Tumor antisense
2516 C > T
A839V
R:TGC > TAC
F:GCA > GTA
2516 C >T
A839V
2525 A >T
N842I
R: GTT > GAT
F: AAC > ATC
2525 A > T
N842I
2587 G > A
G863S
R: ACC > ACT
F : GGT > AGT
2587 G > A
G863S
2587 G > T
G863C
R: ACC > ACA
F: GGT > TGT
2587 G > T
G863C

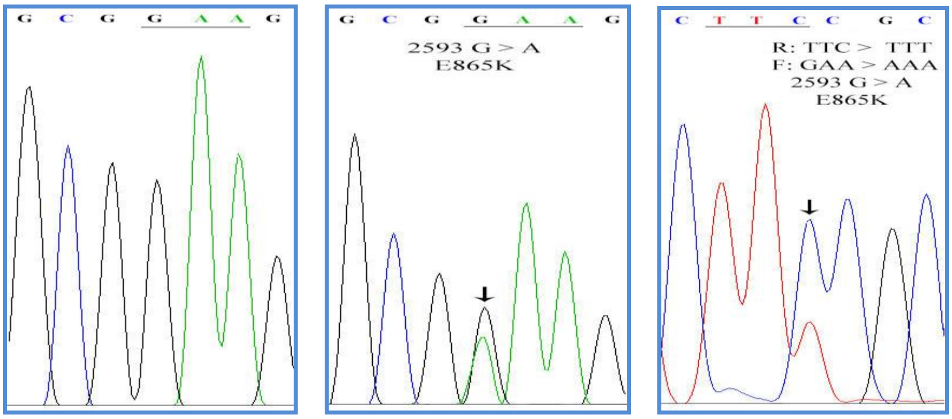

Arrows indicate nucleotide changes. F: forward direction. R: reverse direction.

Supplementary Table 1. Sequences of the primers used for polymerase chain reaction

Gene	Forward primer sequence	Reverse primer sequence
EGFR exon 18	GGCTGAGGTGACCCTTGTC	CTGCGGCCCAGCCCAGAG
EGFR exon 19	AGCATGTGGCACCATCTCAC	GAGAAAAGGTGGGCCTGAGG
EGFR exon 20	ATGCGAAGCCACACTGACGT	TGGCTCCTTATCTCCCCTCC
EGFR exon 21	ATCTGTCCCTCACAGCAGG	TCAGGAAAATGCTGGCTGA

Supplementary Table 2. Mutation pattern and treatment response in 49 pancreatic cancer patients with mutated *EGFR* genes

Mutations			Response rate (%)			Disease control rate (%)		
Nucleotide	Amino acid	No. (%)	G n = 23	G+E n = 26	P	G n = 23	G+E n = 26	P
Exon 18		2 (3%)						
2107C>G	L703V	1 (2%)	0/1 (0%)	-		1/1 (100%)	-	
2116A>G	I706V	1 (2%)	0/1 (0%)	-		0/1 (0%)	-	
Exon 19		23 (37%)						
2183A>G	K728R	12 (19%)	0/4 (0%)	1/8 (13%)	1.000	1/4 (25%)	7/8 (88%)	0.067
2193G>A	W731X	8 (13%)	0/8 (0%)	1/1 (100%)	0.111	1/8 (13%)	1/1 (100%)	0.222
2219T>C	I740T	2 (3%)	-	1/2 (50%)		-	2/2 (100%)	
2270A>G	K757R	1 (2%)	0/1 (0%)	-		1/1 (100%)	-	
Exon 20		31 (50%)						
2300C>A	A767D	1 (2%)	0/1 (0%)	-		0/1 (0%)	-	
2321T>C	V774A	1 (2%)	0/1 (0%)	-		0/1 (0%)	-	-
2333T>C	L778P	15 (24%)	0/8 (0%)	1/7 (14%)	0.467	0/8 (0%)	5/7 (71%)	0.007
2360A>G	Q787R	2 (3%)	-	0/2 (0%)		-	2/2 (100%)	
2399A>G	D800G	1 (2%)	-	0/1 (0%)		-	1/1 (100%)	
2405T>C	V802A	1 (2%)	-	0/1 (0%)		-	1/1 (100%)	
2441T>C	L814P	1 (2%)	-	0/1 (0%)		-	1/1 (100%)	
2462T>C	I821T	9 (15%)	1/4 (25%)	3/5 (60%)	0.524	3/4 (75%)	4/5 (80%)	1.000
Exon 21		6 (10%)						
2516C>T	A839V	1 (2%)	-	1/1 (100%)		-	1/1 (100%)	
2524A>T	N842I	2 (3%)	0/1 (0%)	0/1 (0%)	1.000	0/1 (0%)	1/1 (100%)	1.000
2587G>A	G863S	1 (2%)	0/1 (0%)	-		0/1 (0%)	-	
2587G>T	G863C	1 (2%)	-	0/1 (0%)		-	1/1 (100%)	
2593G>A	E865K	1 (2%)	-	0/1 (0%)		-	1/1 (100%)	
Total		62 (100%)						

Abbreviations: G, gemcitabine alone; G+E, gemcitabine plus erlotinib; X, stop codon.
